# Illustrations of the Effects of Poisons

**Published:** 1834-01-01

**Authors:** 


*Illustrations of the Effects of Poisons.* By GEORGE LEITH ROUPELL, M.D. *The Plates from original Drawings by* ANDREW MELVILLE M'WHINNIE, M.R.C.S. Part I.—London, 1833.

Two of the plates represent the effects produced by large doses of arsenic on the stomachs of a woman, and of a dog; the other two shew the ravages caused in the same way by nitric acid. These plates are of remarkable beauty; and we would point out, as worthy of especial commendation, the true pulpy look given to the mucous membrane of the stomach, in plate ii.

